# Starving for oxygen: the effect of hypoxia on seed germination and secondary dormancy induction in Mediterranean temporary ponds plant species

**DOI:** 10.1111/plb.70148

**Published:** 2025-12-01

**Authors:** M. Di Stefano, C. P. Dominguez, D. Batlla, G. D. Puglia, A. Cristaudo

**Affiliations:** ^1^ Department of Biological, Geological and Environmental Sciences University of Catania Catania Italy; ^2^ Departamento de Producción Vegetal, Facultad de Agronomía, Cátedra de Cultivos Industriales Universidad de Buenos Aires Buenos Aires Argentina; ^3^ Instituto de Fisiología y Ecología Vinculado a la Agricultura (IFEVA) CONICET‐Universidad de Buenos Aires Buenos Aires Argentina; ^4^ Institute for Agriculture and Forestry Systems in the Mediterranean (ISAFoM), National Research Council of Italy (CNR) Catania Italy

**Keywords:** Flooding, germination traits, morpho‐physiological dormancy, near‐anoxia, seed ecophysiology, wetland

## Abstract

- Mediterranean temporary ponds (MTPs) are dynamic habitats where low levels of dissolved oxygen can significantly impact plant life. This study investigated the effect of hypoxia and near‐anoxia on seed germination and the induction of secondary dormancy in 14 plant species, characteristic of this habitat.
- Imbibed seeds were subjected to various oxygen concentrations (0.1, 5, 10, or 21% O_2_), in both light and darkness. We also tested seed ability to recover germination by moving them to aerobic conditions. We measured embryo growth after hypoxic treatments and during recovery in three species with morpho‐physiological dormancy, a rarely investigated response in this dormancy class.
- Our findings revealed a wide range of species‐specific responses. Hypoxia did not inhibit germination in half of the tested species in the light, while near‐anoxia completely inhibited germination in all species. However, most seeds fully recovered germination ability once aerobic conditions were restored. Interestingly, hypoxia in darkness reduced or prevented germination in some species and specifically induced secondary dormancy in *Juncus bufonius*. Surprisingly, seeds of *Bulliarda vaillantii* lost their light requirement for germination under hypoxia. In three *Ranunculus* species with morpho‐physiological dormancy, hypoxia slowed embryo growth, which delayed germination recovery.
- This study reveals that MTPs species have evolved adaptations, ranging from tolerance to hypoxic conditions, to the ability to trigger secondary dormancy, which are crucial to surviving and reproducing in these unique environments. The results offer new insights into the germination ecophysiology of MTPs species and their regeneration niche in temporary wetlands.

## Graphical abstract

This study examined the germination adaptations to hypoxia of species along a plant zonation in Mediterranean temporary ponds, highlighting the key traits that support their survival and regeneration.

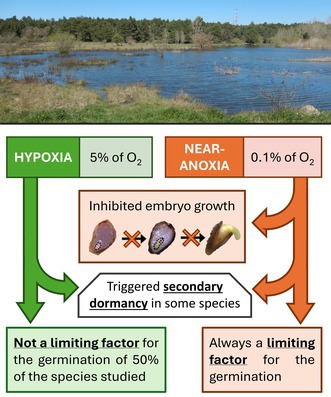

## INTRODUCTION

All plants require water to successfully complete their life cycle, but excess water or flooding significantly alter the gases available for plant physiological and biochemical processes (Loreti *et al*. 2016). In shallow water habitats, dissolved oxygen (DO) fluctuates both daily (Knight *et al*. 2013; Dubuc *et al*. 2017) and seasonally (Wylie & Jones 1987). Mediterranean temporary ponds (MTPs) are a specific type of ephemeral wetland that fill up during the rainy seasons (autumn and winter) and dry out completely during the long dry summer typical of Mediterranean climates (Grillas *et al*. 2004). Outwith the Mediterranean, MTPs are found in South Africa, California, Chile, and Australia, showing similarities, such as the presence of rare pteridophytes (e.g., *Isoetes* spp., *Pilularia* ssp). In MTPs, hypoxic conditions, with DO values < 5 mg L^−1^ (or ppm), are easily reached (Scalici *et al*. 2021; Beccarisi *et al*. 2024).

While adult wetland plants have well‐documented strategies to cope with hypoxia, such as tolerance to prolonged flooding (Crawford 2003), specific adaptations of their seeds and seedlings may not necessarily reflect the life‐history strategies of adult plants (Shipley *et al*. 1989). Germination is, in fact, a crucial stage in a plant's life cycle, and the fine perception of environmental cues, such as DO levels, enables seeds to germinate under the most suitable conditions and time for seedling establishment (Donohue 2005; Corbineau 2022).

Species from well‐drained soils generally fail to germinate under hypoxic conditions, as restricted oxygen availability prevents establishment of seedlings not adapted to cope with low DO levels (Heichel & Day 1972; Leck 1996; Benvenuti 1999; Jarvis & Moore 2008; Corbineau 2022). In contrast, seeds of many wetland species are specifically adapted to germinate under low levels of DO (Baskin & Baskin 2014). For those species, flooded (hypoxic) conditions can either (1) not reduce germinability compared to aerobic conditions (Leck 1996; Prentis *et al*. 2006), or (2) be a requirement for germination (Marler 1969; Coops & Van Der Velde 1995; Leck 1996; Moravcová *et al*. 2001). This positive response to hypoxia ensures that germination can occur even during established flooding, maximizing the colonization capability of wetland species. In this sense, germination responses to oxygen are closely linked to the plant zonation along wetland hydroperiod gradients (Rosbakh *et al*. 2020).

Seeds that can germinate under hypoxic conditions are often also capable of germinating in darkness, a combination of traits that allows germination in deeper parts of ponds or in turbid waters (Baskin & Baskin 2014). For example, germination of *Schoenoplectus lacustris* (L.) Palla is enhanced under hypoxic and dark conditions (Rosbakh *et al*. 2020), which underscores the importance of testing different light conditions when investigating the role of hypoxia in germination of species from different areas of a wetland zonation.

The ability to germinate under anoxia (absence of oxygen) is very rare in nature, having only been reported for a few species (Corbineau & Côme 1995). In fact, anoxic conditions, which can be reached just a few millimetres below the soil surface, can lead to seed death in some species, even after a few days of exposure (Okamoto & Joly 2000; Wiraguna *et al*. 2021). Therefore, wetland species must possess specific adaptations to survive for a long time under these conditions (Kolb & Joly 2010). For instance, anoxia had a protective effect on the seed viability of *Ranunculus baudotii* Godr., but only at a relative humidity of 70% (Carta *et al*. 2018).

Low DO levels not only affect germination but also seed dormancy, which is the innate characteristic that defines the range of environmental conditions under which germination can occur (Finch‐Savage & Leubner‐Metzger 2006). The most common type is physiological dormancy (PD), where an internal physiological mechanism prevents seed germination. Hypoxia can alleviate primary PD in seeds of several species (Tissaoui & Côme 1973; Esashi *et al*. 1976; Corbineau & Côme 1988, 1995). Conversely, in non‐dormant or shallow‐dormant seeds, exposure to hypoxic conditions can trigger secondary dormancy (Lonchamp & Gora 1979; Farmer & Spence 1987; Benvenuti & Macchia 1997; Pekrun *et al*. 1997; Mollard *et al*. 2007; Hoang *et al*. 2013; Phartyal *et al*. 2020). This adaptation may preserve the soil seed bank in temporarily inundated habitats or transiently hypoxic soils (Peralta Ogorek *et al*. 2019).

Another type of seed dormancy is morphological dormancy (MD) (Baskin & Baskin 2014), where seeds have underdeveloped embryos at the time of dispersal that must grow before the radicle can emerge. When MD is combined with PD, the seeds have morpho‐physiological dormancy (MPD). Species with MPD inhabit temporarily inundated wetlands (Carta *et al*. 2012; Di Stefano *et al*. 2025), meaning their underdeveloped embryo can experience hypoxia before germination. It has been speculated that hypoxia could slow down the regular seed development processes through a decrease in respiration (Rolletschek *et al*. 2024), but whether low oxygen levels can inhibit embryo growth in species with MPD is still unknown.

Few studies have analysed the germination ecophysiology of species inhabiting MTPs (Carta 2016; Puglia *et al*. 2018; Di Stefano *et al*. 2025), focusing on light, temperature, and various pre‐treatments. To date, only one study has analysed the role of oxygen in seed survival for a single MTPs species from the Mediterranean (Carta *et al*. 2018). Thus, investigating the germination response to low O_2_ levels in species from MTPs could shed light on possible adaptations of these highly specialized plants. For example, Bliss & Zedler (1997) highlighted the importance of flooded (hypoxic) conditions for the germination of Californian vernal pool species, a phenomenon that affects community structure. Hypoxia was even a germination stimulus for two Californian vernal pool species (Keeley 1988).

Given these considerations, this study aimed to address two main questions: (1) what is the effect of hypoxia and near‐anoxia on the germination of MTPs species with PD and MPD; and (2) do low oxygen levels trigger the induction of secondary dormancy in these seeds?

## MATERIAL AND METHODS

### Study species, seed collection, and storage

To understand the role of low oxygen levels on the seed germination and dormancy of MTPs species, we selected 14 ephemeral plants (Table S1) that characterize the communities of MTPs in Sicily (Italy), according to numerous vegetation surveys carried out in this region (Sciandrello *et al*. 2016; Brullo *et al*. 2022). The type of dormancy was inferred or obtained, when possible, from Di Stefano *et al*. (2025) and previous experiments conducted on the same seed lots as in this study. Species differed in functional groups according to their morphological response to temporary flooding *sensu* Brock & Casanova (1997). This characterizes three areas in the small‐scale plant zonation typical of MTPs (Bauder 2000; Rocarpin *et al*. 2016; Lanfranco *et al*. 2020): long‐lasting flooded areas, short‐lasting flooded areas, and the outer belt of the pond.

Mature dispersal units (hereafter referred to as ‘seeds’) of the selected species were harvested at the time of their natural dispersal in different MTPs of the Hyblean plateau, near the villages of Buccheri and Villasmundo (south‐eastern Sicily; Table S1). Seed collection of each species involved over 200 randomly selected plants. Seeds were cleaned and stored at 20°C with a controlled relative humidity of 40%. This storage simulated after‐ripening in the soil, which in some MTPs species is necessary to overcome PD, if present (Carta 2016; Di Stefano *et al*. 2025). Seed germination experiments started in September 2024.

### Seed germination under different oxygen concentrations and light conditions

To test the effect of low oxygen levels on germination percentages, a common constant temperature (15°C) was chosen to simulate the buffering effect of inundation on daily temperature fluctuations. Germination was tested under alternating light/dark conditions (L/D; photoperiod 12 h/12 h) at four different oxygen concentrations: 0.1% (near‐anoxia), 5%, 10% (hypoxia), and 21% (air). To examine the influence of darkness (D, photoperiod 0/24 h) in combination with hypoxia, seeds were incubated at 15°C at 5% and 21% O_2_. Each germination test lasted 14 days. Germination was scored daily for seeds incubated in air, and at the end of the test (Day 14) for seeds incubated under near‐anoxia/hypoxia. Germination was defined as the protrusion of the radicle through the seed coat for half of the seed's total length.

The desired stable near‐anoxic and hypoxic environment was achieved following Dominguez *et al*. (2019). A transparent semi‐sealable plastic subchamber (35.56 cm width × 30.48 cm depth × 15.24 cm height, model C‐274; BioSpherix, USA) coupled with an oxygen controller (PRO‐OX model 110, BioSpherix) was used. The oxygen controller both detected the oxygen concentration inside the subchamber and supplied nitrogen to achieve and maintain the desired oxygen concentration below 21% (Fig. S1a). The subchamber was placed inside a germination incubator (MIR‐253, Sanyo, Japan). In contrast, the O_2_ concentration of 21% (aerobic condition) was achieved by placing pseudo‐replicates inside the incubator, outside the subchamber. In L/D, a test in air was performed together with each hypoxic condition tested. Since no statistically significant differences were found in final germination percentages (FGP) in all the tests performed at 21% under L/D for each species, only the results of the control in air carried out together with 5% O_2_ were used for subsequent analyses.

For each incubation treatment (i.e., combinations of oxygen concentration and light condition), three pseudo‐replicates of 30 seeds were sown in plastic boxes filled with 25 mL 1% agar. Rectangular plastic boxes (5.5 cm width × 4 cm depth × 2.5 cm high) were used to optimize the space inside the subchamber. To facilitate gas exchange, three holes of 6 mm in diameter were made in the cap of each box (Fig. S1b).

### Germination response following recovery of aerobic conditions

To test the seed ability to recover germination (i.e., lack of induction into secondary dormancy), ungerminated seeds at the end of each oxygen treatment were moved to aerobic conditions for an additional 2 weeks (from Day 14 to Day 28). The recovery condition (21% of O_2_, 15°C, light with a photoperiod of 24 h) was achieved by shifting the seeds to another incubator (MIR‐253, Sanyo, Japan). Germination during the recovery test in air was checked daily. The viability of seeds that failed to germinate even after the recovery test was assessed using the tetrazolium test. Table 1 provides an overview of the experimental design adopted in this study.

**Table 1:** Overview of the experimental design adopted in this study.

Temperature	Light regime	Oxygen concentration (Day 1–14)	Recovery condition (Day 14–28)
15°C	L/D	0.1%	15°C; L; 21% O_2_
		5%	
		10%	
		21%	
	D	5%	
		21%	

L/D: alternating light regime (photoperiod 12 h/12 h); D: darkness (photoperiod 0 h/24 h); L: light (photoperiod 24 h).

### Embryo growth in air after exposure to hypoxic and near‐anoxic conditions

To analyse the role of low oxygen levels and light on embryo growth, we used seeds of three MTPs species from the genus *Ranunculus* that have morpho‐physiological dormancy (MPD): *Ranunculus ophioglossifolius*, *R. lateriflorus*, and *R. sardous*. For two of them, namely *R. ophioglossifolius* and *R. lateriflorus*, it was found that embryo growth occurs rapidly if seeds are imbibed at 15°C in the light (Di Stefano *et al*. 2025). Seeds of these species were thus placed at 0.1%, 10%, and 21% O_2_ at 15°C in L/D for 2 weeks (14 days). After this period, seeds were moved to recovery conditions in air for 9 days (from Day 14 to Day 23). Observations on embryo elongation were made after 24 h of imbibition (Day 0), after the 2‐week oxygen treatments (Day 14), and after 3, 6, and 9 days of the recovery test (Day 17, 20, and 23). At each time point, 10 seeds of each species from each treatment were sampled and cut in half with a scalpel under a stereomicroscope (Leica MZ6). Wet filter paper was used to avoid desiccation of the sample during this process. Images were captured using an MS60 (Mshot) camera. MShot Image Analysis System v. 1.0 was used to measure embryo and seed length. To determine the critical E:S ratio, 100 additional seeds were incubated at 15°C under light/dark conditions, and the critical E:S ratio averaged from 10 seeds with split seed coats but no visible radicle protrusion. Values coinciding with the critical E:S were assigned to seeds that surpassed it.

### Statistical analysis

All analyses were performed in R v. 4.4.3 (R Core Team 2025). To calculate FGP at the end of each test, generalized linear models (GLMs, binomial error distribution, logit link function) were fitted including species, O_2_ concentration, and their interactions as explanatory factors. Two different GLMs were fitted with the results obtained in L/D and D, because of the different O_2_ concentrations tested in these two experiments. FGP and standard errors (SE) were extracted from the models as Estimated Marginal Means (EMMs) with the ‘emmeans’ package (Lenth *et al*. 2019). Germination time courses were obtained by fitting log‐logistic curves on the cumulative proportions of germinated seeds with the ‘drmte’ function of the ‘drcte’ package (Onofri *et al*. 2022).

## RESULTS

### Effects of oxygen availability under different light conditions on seed germination and subsequent germination recovery in air

**Fig. 1:**
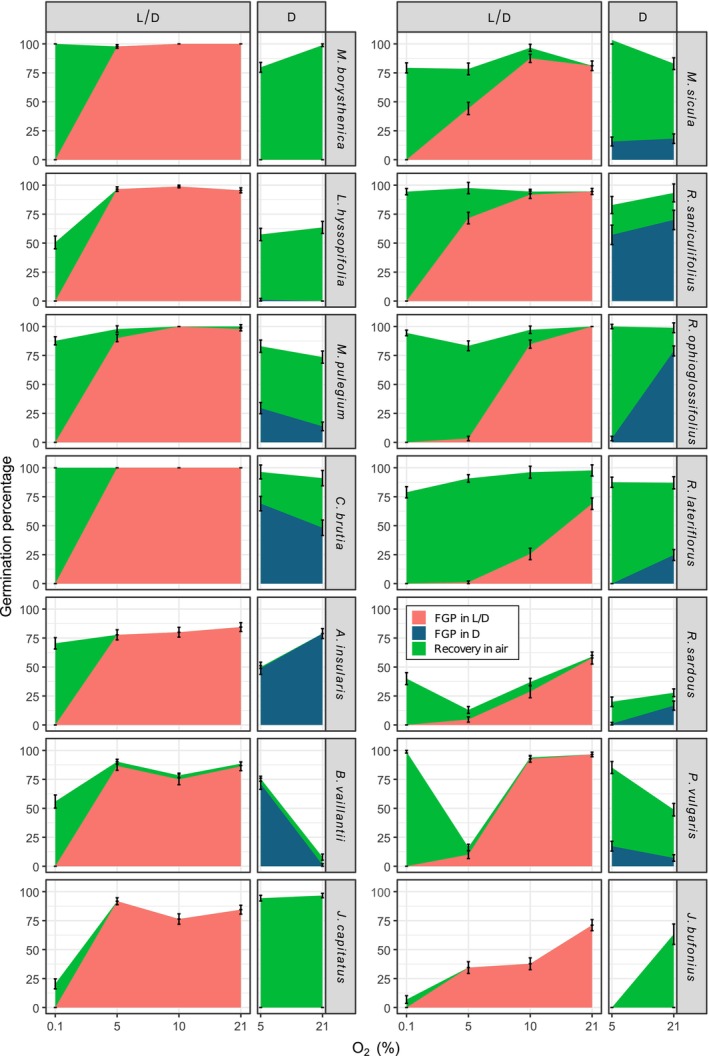
Final germination percentage (FGP) under hypoxia and near‐anoxia, under different light regimes, and during recovery in aerobic conditions of seeds of 14 MTPs plant species. Red areas represent germination in light (L/D; 12 h/12 h photoperiod) for seeds exposed to near anoxia (0.1% O_2_), hypoxia (5% and 10% O_2_), and standard aerobic conditions (21% O_2_). Blue areas show germination achieved under hypoxia (5% O_2_) and under air (21% O_2_) in complete darkness (D). Seeds were incubated at 15°C and tests lasted 2 weeks. Green areas indicate germination percentage at the end of the recovery test in air (21% O_2_; 15°C; light with a photoperiod of 24 h for additional 2 weeks). Vertical bars represent standard error (SE).

Hypoxia (5% and 10% O_2_) was not a limiting factor for the germination of half of the 14 tested species (e.g., *Antinoria insularis*, *Callitriche brutia*, *Bulliarda vaillantii*, *Lythrum hyssopifolia*, *Middendorfia borystenicha*, *Mentha pulegium*, *Juncus capitatus*; red areas in left column of species in Fig. 1) as no statistically significant differences were detected with FGPs achieved in air (*p* > 0.12 in all cases). Near‐anoxia (0.1% O_2_), on the other hand, completely inhibited germination in all species (FGP = 0%). In *Myosotis sicula, Ranunculus saniculifolius*, and *Pulicaria vulgaris*, 5% O_2_ resulted in lower germination than in 10% O_2_ (*p* < 0.005 in all cases), while 10% O_2_ did not decrease the FGPs compared to air (*p* > 0.64 in all cases). In *Juncus bufonius*, the two hypoxic levels tested (5% and 10%) differed from the control but not between them (*p <* 0.001 and *p* = 0.97, respectively). In *R. ophioglossifolius, R. lateriflorus*, and *R. sardous*, germination decreased with O_2_ concentration.

**Fig. 2:**
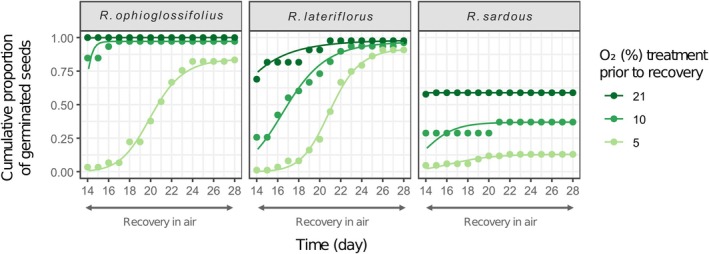
The effect of hypoxic treatments on germination recovery in air in three species of the genus *Ranunculus*. Germination time course of *R. ophioglossifolius*, *R. lateriflorus*, and *R. sardous* during a 2‐week recovery test in air (21% O_2_, 15°C, light with a photoperiod of 24 h), following an initial 2‐week incubation (Day 1–14) under hypoxic (5% and 10% O_2_) and aerobic (21% O_2_) conditions at 15°C in the light (12 h/12 h photoperiod). This figure shows the recovery of germination from Day 14 to Day 28.

**Fig. 3:**
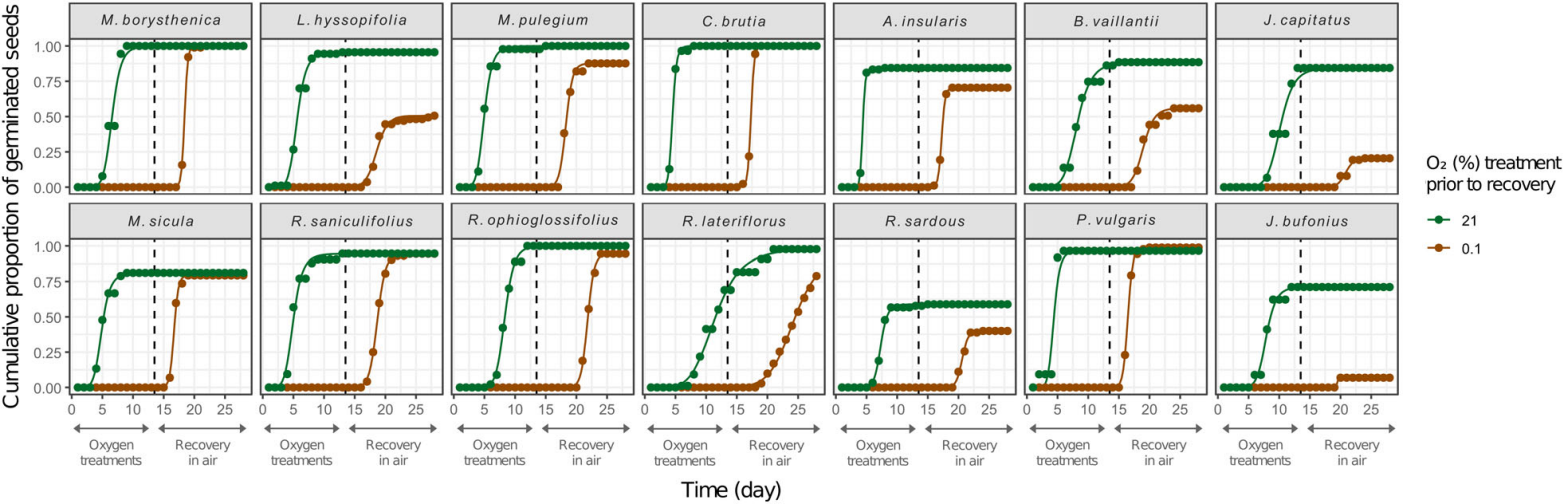
Effect of the near‐anoxic treatment on seed germination dynamics of 14 MTPs plant species. Germination time course during the initial 2‐week oxygen treatment (Days 1–14) under near‐anoxia (0.1% O_2_, brown lines) and aerobic (21% O_2_, green lines) conditions at 15°C in the light (12 h/12 h photoperiod). This was followed by a 2‐week recovery period (Days 14–28) in air (21% O_2_) at 15°C under light with a photoperiod of 24 h.

Shifting the ungerminated seeds to recovery conditions in air (15°C, light, 21% O_2_) for 2 additional weeks provoked different germination behaviours. Many species in which hypoxia decreased FGPs recovered germination when moved to air (Fig. 1, green areas). On the other hand, seeds of *P. vulgaris* previously incubated at 5% for 2 weeks failed to fully recover germination, as did the seeds of *J. bufonius* and *R. sardous* from both 5% and 10% O_2_. Regarding FGPs, 2 weeks of near‐anoxia still allowed most of the species to fully recover germination (reaching FGPs similar to those in air at the end of the recovery test), with the exceptions of *J. bufonius* and *J. capitatus* and, to a lesser extent, *B. vaillantii* and *L. hyssopifolia*. In *R. ophioglossifolius, R. lateriflorus*, and *R. sardous*, decreasing O_2_ also resulted in a forward temporal shift of the germination curves when seeds were moved to air, particularly noteworthy in the case of *R. lateriflorus* (Fig. 2). Moreover, ungerminated seeds under near‐anoxia, when moved to recovery conditions in air, showed the same germination dynamic (starting day and slope of the curve) as seeds that never experienced near‐anoxia, despite reaching different final germination percentages in some cases, as explained (Fig. 3). All the ungerminated seeds at the end of the recovery test were found viable in the tetrazolium test.

Germination under hypoxia in darkness was species‐specific. Darkness, regardless of the oxygen level, indeed inhibited germination in species from the Juncaceae and Lythraceae families (*L. hyssopifolia*, *M. borystenicha*, *J. bufonius*, and *J. capitatus*; blue areas of Fig. 1). In contrast, hypoxia eliminated the light requirement for seed germination in *B. vaillantii* seeds (71.11% in hypoxia against 0% in air in darkness), and significantly increased germination compared to air in *M. pulegium* and *C. brutia* (*p <* 0.05 in both species). On the other hand, germination was significantly reduced by hypoxia in darkness in *A. insularis*, *R. ophioglossifolius*, *R. lateriflorus*, and *R. sardous* (*p <* 0.001 in each case). In other species, no significant differences were scored between FGPs achieved at 5% and 21%. This is the case for *R. saniculifolius* (*p =* 0.28), *M. sicula* (*p =* 0.66), and *P. vulgaris* (*p =* 0.05).

The response of ungerminated seeds in darkness to the recovery condition also varied among species (green areas of Fig. 1). *J. capitatus* and *R. ophioglossifolius* were the only two species that fully recovered germination (FGP close to 100%) regardless of oxygen level. In contrast, seeds of *J. bufonius* coming from the hypoxic environment failed to recover germination when moved to light, and seeds of *M. borysthenica* previously incubated under hypoxia recovered germination to a lower percentage (*p* < 0.001) than those incubated in air. Ungerminated seeds of some species failed to fully recover germination under both a hypoxic and an aerobic environment. This was the case for *L. hyssopifolia*, *M. pulegium*, *R. lateriflorus*, *R. saniculifolius*, and *R. sardous* that, despite having recovered germination with comparable cumulative FGPs between hypoxia and air, still contained ungerminated fractions at the end of the recovery test. Ungerminated seeds of *A. insularis*, *B. vaillantii*, and *R. sardous* showed little or no germination recovery (FGP < 6.74% in all cases) when moved to light at both 5% and 21% O_2_. On the other hand, seeds of *M. sicula* and *P. vulgaris* that experienced hypoxia recovered germination with statistically higher FGPs than those previously exposed to air (*p* < 0.001 in both cases). Also, in this case, the ungerminated seeds at the end of the recovery test were found viable with the tetrazolium test.

### Embryo growth in air after exposure to hypoxic and near‐anoxic conditions

**Fig. 4:**
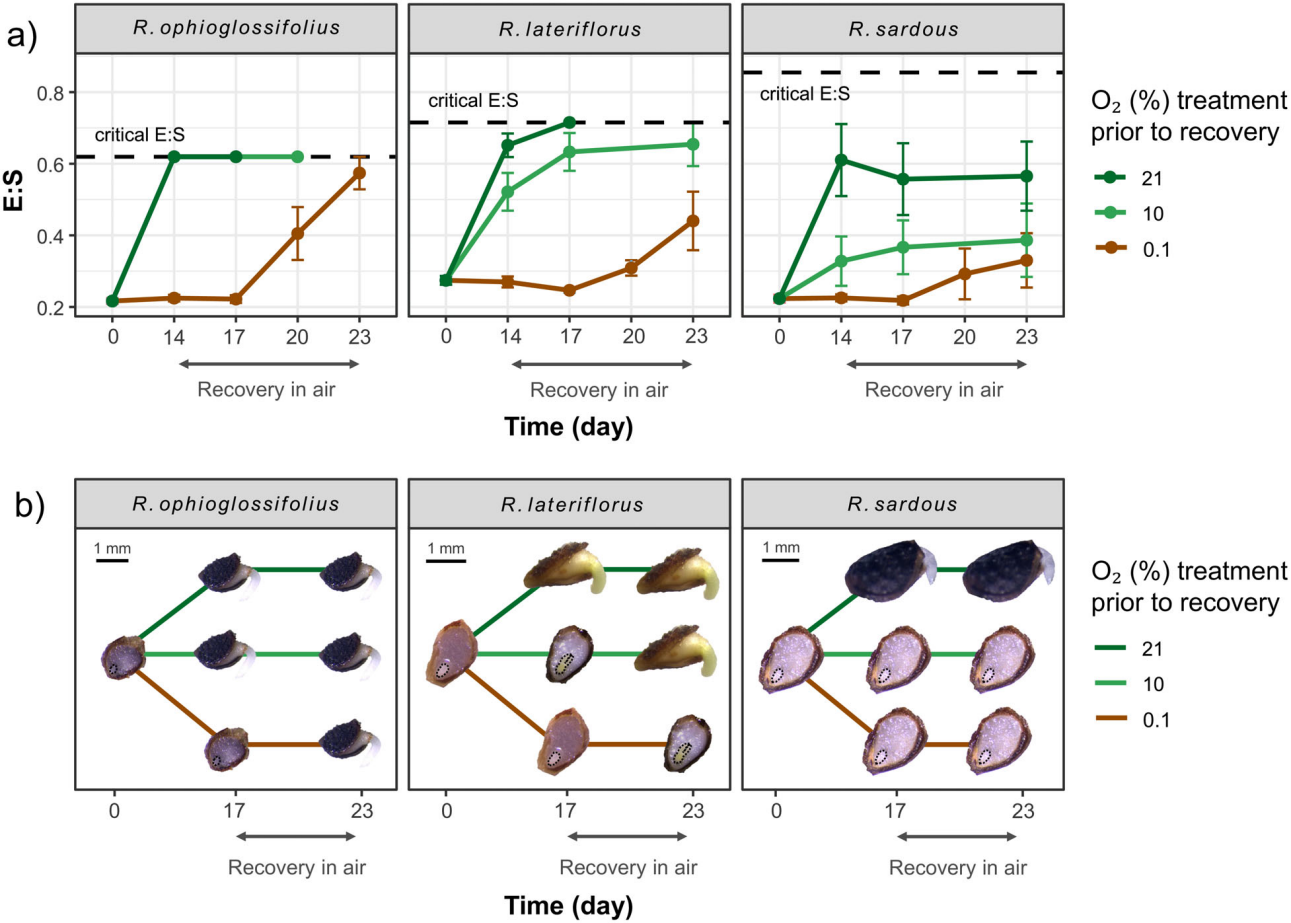
The effect of hypoxia and near‐anoxia on embryo growth in three *Ranunculus* species with morpho‐physiological dormancy. (a) Mean embryo‐to‐seed length ratio (E:S ratio ± SE) for seeds at the start of the experiment (Day 0), after 2 weeks of treatments (Day 14) under near‐anoxia (0.1% O_2_), hypoxia (10% O_2_), or aerobic (21% O_2_) conditions (15°C; 12 h/12 h photoperiod), and after 3, 6, and 9 days (Days 17, 20, and 23) of recovery in air (21% O_2_; 15°C; light with a photoperiod of 24 h). (b) Selection of longitudinal sections showing embryo growth after 3 and 9 days (Days 17 and 23) of recovery in air.

Available oxygen influenced the embryo growth in the three studied *Ranunculus* species in L/D (Fig. 4a). Air (21%) combined with light allowed seeds of *R. ophioglossifolius* to achieve or exceed the critical E:S at the end of the test (Day 14), while for *R. lateriflorus* seeds this happened after 6 days of recovery test (Day 17). Seeds of *R. sardous*, on the other hand, did not reach the critical E:S as a significant fraction of ungrown embryos were found even after 9 days under recovery conditions (Day 23). Under near‐anoxia, embryo growth was completely inhibited in all species, as was clear from embryo sizes after 2 weeks at 0.01% (Day 14; Fig. 4b). An increase in E:S was observed in the three species only after 6 days (Day 20) of germination recovery in air. Regarding the hypoxic concentration of 10%, this allowed embryo elongation but acted differently depending on species. *R. ophioglossifolius* was the only species in which 10% O_2_ resulted in seeds reaching the critical E:S at the end of the treatment (Day 14), with no difference compared to results observed in air. In *R. lateriflorus* and *R. sardous*, however, 2 weeks at 10% limited the embryo growth. In these species, the E:S at 10% was always lower than in air at any sampling interval.

## DISCUSSION

For half of the tested Mediterranean temporary ponds (MTPs) species, oxygen concentrations of 5% and 10% (hypoxia) were not limiting for germination in the light, as seeds reached the same percentages as in air (21% O_2_). This high germination response to hypoxic conditions highlights an adaptation of MTPs species to promptly germinate even under hypoxic water, if they perceive light. This behaviour has also been found in other wetland species (Baskin & Baskin 2014). In MTPs, this adaptation would allow emerging seedlings to escape competition, as germination of more opportunistic terrestrial species is severely limited by hypoxia (Heichel & Day 1972; Benvenuti 1999; Corbineau 2022). The results obtained with hypoxia in the light also confirmed that *J. bufonius* and *L. hyssopifolia* do not require strict aerobic conditions to germinate, as reported by Bliss & Zedler (1997), Phartyal *et al*. (2020) and Rosbakh *et al*. (2020). On the other hand, our population of *M. pulegium* seems to be insensitive to hypoxia, both in light and in dark conditions, in contrast to observations of Phartyal *et al*. (2020) and Rosbakh *et al*. (2020) using different incubation temperatures in populations from mudflats.

Furthermore, in most of the studied species, hypoxia did not induce secondary dormancy. In temporary wetlands, such as MTPs, this adaptation could result in a competitive advantage. Given the high variability of flooding events and their duration, species that germinate poorly under flooding can promptly germinate if water suddenly recedes. In seeds of *J. bufonius*, *P. vulgaris*, and *R. sardous*, however, hypoxia lowered germination, and the seeds failed to fully recover germination ability when shifted to air, indicating the induction of secondary dormancy after hypoxia. The behaviour of these latter three species might limit their spatial germination niche to never or only briefly inundated areas of MTPs, where hypoxia may not be perceived by the seeds.

In this sense, *R. sardous* and *P. vulgaris* characterize the seldom inundated outer belt or slight depressions of MTPs (Ernandes & Marchiori 2013; Nimis *et al*. 2014), while *J. bufonius* is a widespread species (POWO 2025) also found in other habitats characterized by moist/waterlogged soils (Van Loenhoud & Sterk 1976; Cope & Stace 1978). In contrast, species colonizing long‐lasting flooded areas of MTPs, such as *C. brutia*, demonstrate adaptations in their seeds to tolerate hypoxic conditions, likely reflecting frequent and prolonged oxygen‐deficient exposure (Rosbakh *et al*. 2020). The perennial *M. pulegium* can germinate across different oxygen gradients, probably because it can rely also on vegetative persistence together with seed recruitment. Finally, some small‐sized species restricted to short‐lasting flooded areas, such as *A*. *insularis*, *B*. *vaillantii*, *L*. *hyssopifolia, M*. *borystenicha*, and *J. capitatus*, can also germinate under hypoxia. Although the adult plants are not adapted to live under deep and prolonged submergence, the seeds of these species appear to be ready to germinate even if shallow inundation can result in hypoxic conditions in the sediment of MTPs. In this sense, hypoxia can be achieved at less than 2 cm below the surface in lake sediments (Bonnewell *et al*. 1983). Together, these contrasting seed ecology strategies highlight how oxygen tolerance contributes to species zonation along hydroperiod gradients in wetland systems (Rosbakh *et al*. 2020).

Near‐anoxia (0.1% O_2_), on the other hand, completely inhibited germination in all the tested species. The inhibitory effect of anoxia ensures that germination is avoided in case of prolonged, severe flooding of the ponds. This is a common adaptation in plants, with germination under anoxia being reported for very few species (Corbineau & Côme 1995). But seeds preserved viability after 2 weeks under near‐anoxic conditions. In flooded soil, the oxygen level can be less than 1%, due to a strong reduction in its diffusion in water with respect to air (Gambrell *et al*. 1991). Thus, this tolerance of seeds of MTPs species to anoxia is of fundamental importance in maintaining a rich and viable soil seed bank even in cases of severe anoxic events. Furthermore, near‐anoxia did not induce secondary dormancy or modify the germination dynamics of seeds when moved to air in most of the studied species. This adaptation ensures that germination could occur after a temporary anoxic event. The high fluctuations in dissolved oxygen (DO) typical of temporary wetlands (Scholnick 1994) would not cause seeds of most of the species to become dormant, maximizing their colonization capacity when the stressful factor disappears. On the other hand, near‐anoxia induced secondary dormancy in seeds of the two studied *Juncus* species. Seeds of these species would, therefore, remain dormant in the soil seed bank after an anoxic event, as noted by Benvenuti & Macchia (1995) for *Datura stramonium* L.

The germination response to hypoxia in darkness was species‐specific. Dark and hypoxic conditions can be achieved, for example, if seeds are buried in the soil during flooding. Surprisingly, seeds of *B. vaillantii* lost their light requirement when incubated under hypoxia. This unique adaptation seems to be a flooding‐sensing mechanism that allows *B. vaillantii* to massively colonize the ponds after the fill. Furthermore, a hypoxic and dark environment clearly triggered secondary dormancy in seeds of *J. bufonius*, allowing the maintenance of a rich soil seed bank in case of prolonged hypoxia perceived by buried seeds. In this species, hypoxia in the light also led to secondary dormancy, but only in a fraction of the seeds incubated in this condition. Moreover, in seeds of most of the studied species, darkness inhibited germination regardless of the oxygen level. This is a common trait in MTPs species (Carta 2016) that prevents seed germination from taking place underwater or deep within the soil (Baskin & Baskin 2014).

In three *Ranunculus* species with MPD, namely *R. ophioglossifolius*, *R. lateriflorus*, and *R. sardous*, hypoxia lowered the germination performance. This is related to a slowing of embryo growth inside the seeds during the low oxygen treatments. A possible explanation can be traced to the role of oxygen in the early steps of embryo growth, so at lower DO, the pace of seed metabolism can be reduced (Bewley 1997). As far as we know, this is the first study that has assessed the role of hypoxia and near‐anoxia in inhibiting or suppressing embryo growth of species with MPD. Furthermore, at the hypoxic level of 10% oxygen, *R. ophioglossifolius* germinated with higher percentages than *R. lateriflorus*. This confirms the differentiation of the seed germination niche between these two species that was pointed out by Di Stefano *et al*. (2025), with *R. ophioglossifolius* also being adapted to temperate, permanent wetlands of north‐west Europe. In *R. saniculifolius*, hypoxia was not a limiting factor for germination, resulting in only a slight decrease in percentages under 5% oxygen compared to air. This species, in contrast to the other three *Ranunculus* species mentioned above, belongs to a different subgenus: *Batrachium* (DC) A. Grey (Johansson 1998). This subgenus includes species highly adapted to the aquatic environment, with *R. saniculifolius* showing a high degree of phenotypic and germination plasticity to deal with dry or low‐rainfall periods (Fernández‐Zamudio *et al*. 2021). Thus, the embryo of this species, as well as the seeds of other aquatic species, may have evolved a mechanism to tolerate low oxygen levels, allowing germination to resume when more favourable conditions return. This adaptation could be a key strategy for survival in habitats characterized by strong fluctuations in dissolved oxygen levels, providing a significant ecological advantage in highly unpredictable environments like MTPs. In contrast, embryo growth is prevented in seeds of *R. sardous* exposed to two hypoxic treatments (5% and 10% O_2_) when aerobic conditions were restored. As discussed above, this trait confines germination of this species to the areas of the ponds with wet/waterlogged soils.

Our findings demonstrate that plant species from MTPs have evolved a wide range of germination strategies to cope with fluctuating oxygen availability, from tolerance to hypoxia, to the induction of secondary dormancy. We specifically showed that a decrease in DO significantly slows or completely inhibits embryo growth in species with morpho‐physiological dormancy (MPD). These results provide novel insights into the ecophysiological mechanisms that enable MTP species to persist in this dynamic and challenging habitat. This emphasizes once again the importance of integrating germination traits in studies on the ecological dynamics of plant communities (Jiménez‐Alfaro *et al*. 2016).

The vulnerability of MTPs to climate change, particularly the increasing unpredictability of precipitation patterns (Zacharias & Zamparas 2010), underscores the importance of our findings. The ability to predict species resilience to abrupt changes in DO levels, such as those caused by flash flood events projected to become more frequent in the Mediterranean region (Kyselý *et al*. 2012), is crucial. A better understanding of the physiological responses of seeds to hypoxia and their survival mechanisms is therefore paramount for developing effective conservation and management strategies for these highly threatened wetland ecosystems.

## Author Contributions

MDS, GDP, and AC conceived the study. MDS, CPD, and DB performed the seed germination experiments. MDS analyzed the data and led the writing process. GDP, DB, and AC supervised the research. AC acquired the funds. All authors helped in critically revising the manuscript and gave final approval for publication.

## Supporting information


**Table S1.** List of the 14 selected MTPs species. For each species, information about the dormancy type, the functional group, the zone colonized in the MTPs zonation, the life cycle, the collecting site, and date is given. Dormancy type: physiological dormancy (PD); morphophysiological dormancy (MPD). Functional groups (sensu Brock & Casanova 1997): fluctuation‐responders with floating leaves (fr‐fl); fluctuation‐responders with heterophylly (fr‐h); fluctuation‐tolerators with small size (ft‐s); terrestrial species from damp places (ter‐d). Area colonised in MTPs: long‐lasting flooded areas (LL); short‐lasting flooded areas (SL); outer belt (OB). Nomenclature followed Pignatti *et al*. (2019).
**Fig. S1.** Equipment used to perform the low oxygen experiments. (a) Schematical representation of the equipment's setup used to achieve stable hypoxic and near‐anoxic conditions (from BioSpherix, modified); (b) plastic boxes used for the experiments: three holes (6 mm in diameter) were made in each cap to facilitate gas exchange inside the subchamber.
