# Supplementary material for: Starving for oxygen: the effect of hypoxia on seed germination and secondary dormancy induction in Mediterranean temporary ponds plant species
Source: Plant Biol (Stuttg). 2025 Dec 1;28(2):432–40. doi: 10.1111/plb.70148 (PMC12884031; doi:10.1111/plb.70148)
Supplement: Supplementary file 1 — Table S1. List of the 14 selected MTPs species. For each species, information about the dormancy type, the functional group, the zone colonized in the MTPs zonation, the life cycle, the collecting site, and date is given. Dormancy type: physiological dormancy (PD); morphophysiological dormancy (MPD). Functional groups (sensu Brock & Casanova 1997): fluctuation‐responders with floating leaves (fr‐fl); fluctuation‐responders with heterophylly (fr‐h); fluctuation‐tolerators with small size (ft‐s); terrestrial species from damp places (ter‐d). Area colonised in MTPs: long‐lasting flooded areas (LL); short‐lasting flooded areas (SL); outer belt (OB). Nomenclature followed Pignatti et al. (2019). Fig. S1. Equipment used to perform the low oxygen experiments. (a) Schematical representation of the equipment's setup used to achieve stable hypoxic and near‐anoxic conditions (from BioSpherix, modified); (b) plastic boxes used for the experiments: three holes (6 mm in diameter) were made in each cap to facilitate gas exchange inside the subchamber. [file PLB-28-432-s001.doc]

**Table S1** – List of the 14 selected MTPs species. For each species, information about the dormancy type, the functional group, the zone colonized in the MTPs zonation, the life cycle, the collecting site, and date is given. Dormancy type: physiological dormancy (PD); morphophysiological dormancy (MPD). Functional groups (sensu Brock & Casanova, 1997): fluctuation-responders with floating leaves (fr-fl); fluctuation-responders with heterophylly (fr-h); fluctuation-tolerators with small size (ft-s); terrestrial species from damp places (ter-d). Area colonised in MTPs: long-lasting flooded areas (LL); short-lasting flooded areas (SL); outer belt (OB). Nomenclature followed Pignatti et al. (2019).

| **Species** | **Family** | **Dormancy type** | **Functional group** | **Area** | **Lyfe cycle** | **Seed collecting site** | **Seed collecting date** |
| --- | --- | --- | --- | --- | --- | --- | --- |
| *Antinoria insularis* | Poaceae | PD | ft-s | SL | Annual | Contessa di Sopra, Buccheri | July, 2023 |
| *Bulliarda vaillantii* | Crassulaceae | PD | ft-s | SL | Annual | Cozzo tre grotte, Buccheri | May, 2024 |
| *Callitriche brutia* | Plantaginaceae | PD | fr-h | LL | Annual | Cozzo tre grotte, Buccheri | June, 2023 |
| *Juncus bufonius* | Juncaceae | PD | ft-s | SL | Annual | Contessa di Sopra, Buccheri | July, 2023 |
| *Juncus capitatus* | Juncaceae | - | ft-s | SL | Annual | Contessa di Sopra, Buccheri | July, 2023 |
| *Lythrum hyssopifolia* | Lythraceae | - | ft-s | SL | Annual | Contessa di Sopra, Buccheri | July, 2023 |
| *Mentha pulegium* | Lamiaceae | - | ter-d | OB | Perennial | Contessa di Sopra, Buccheri | August, 2023 |
| *Middendorfia borysthenica* | Lythraceae | - | ft-s | SL | Annual | Contessa di Sopra, Buccheri | July, 2023 |
| *Myosotis sicula* | Boraginaceae | PD | ft-s | SL | Annual | Contessa di Sopra, Buccheri | July, 2023 |
| *Pulicaria vulgaris* | Asteraceae | - | ter-d | OB | Annual | Contessa di Sopra, Buccheri | August, 2023 |
| *Ranunculus lateriflorus* | Ranunculaceae | MPD | fr-fl | LL | Annual | Tenuta corvo, Villasmundo | May, 2022 |
| *Ranunculus ophioglossifolius* | Ranunculaceae | MPD | fr-fl | LL | Annual | Contessa di Sopra, Buccheri | July, 2023 |
| *Ranunculus saniculifolius* | Ranunculaceae | MPD | fr-h | LL | Annual | Cozzo tre grotte, Buccheri | June, 2023 |
| *Ranunculus sardous* | Ranunculaceae | MPD | ter-d | OB | Annual | Tenuta Corvo, Villasmundo | June, 2023 |

| 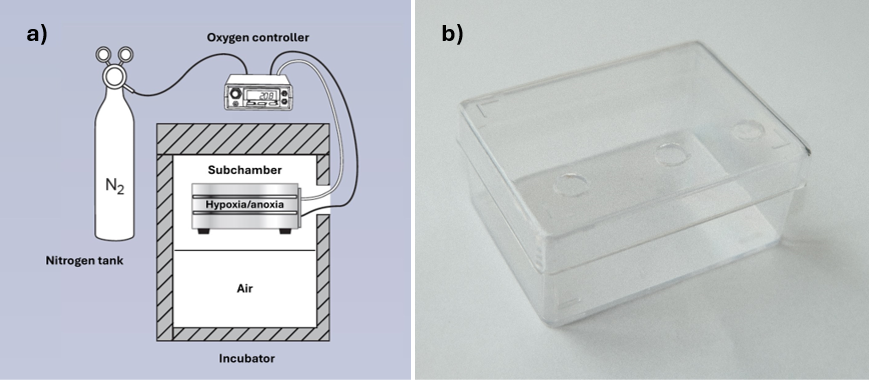 |
| --- |
| **Fig. S1** – Equipment used to perform the low oxygen experiments. a) Schematical representation of the equipment’s setup used to achieve stable hypoxic and near-anoxic conditions (from BioSpherix, modified); b) plastic boxes used for the experiments: three holes (6 mm in diameter) were made in each cap to facilitate gas exchange inside the subchamber. |
